# Coordinating influence of multilayer graphene and spherical SnAgCu for improving tribological properties of a 20CrMnTi material

**DOI:** 10.1039/c7ra12756a

**Published:** 2018-04-17

**Authors:** Xiaoxue Li, Jingli Xu

**Affiliations:** School of Mechanical and Electronic Engineering, Wuhan University of Technology 122 Luoshi Road Wuhan 430070 China xujl1996@163.com; School of Mechanical and Traffic Engineering, Ordos Institute of Technology 1 Ordos Avenue East Ordos 017000 China

## Abstract

In order to increase the service life and operational reliability of a 20CrMnTi-steel-based gearing system, the friction and wear behavior of 20CrMnTi needs to be further improved. In this study, the sliding friction and wear properties of 20CrMnTi, 20CrMnTi-1.50 wt% graphene (20-Gr), 20CrMnTi-15.00 wt% SnAgCu (20-Sn), and 20CrMnTi-15.00 wt% SnAgCu-1.50 wt% graphene (20-Gr-Sn) were examined on a ball-on-disk tribometer. The friction and wear properties at 0–85 min of 20-Gr-Sn were significantly better compared to those of 20CrMnTi, 20-Gr, and 20-Sn. Metallic oxides appeared on the smooth wear scar of 20-Gr-Sn, which were tightly combined with the 20CrMnTi-based material. This caused a lubrication film with low hardness (approximately 1.25 GPa) to form on the grain-refined layer with high hardness (approximately 5.92 GPa). Graphene and SnAgCu in the lubrication film exhibited excellent coordinating lubrication to result in a low friction coefficient and lower wear rate. The obtained results can provide a good reference for increasing the service life of 20CrMnTi-steel-based gear systems.

## Abstract 2

In order to increase the service life and operational reliability of a 20CrMnTi-steel-based gearing system, the friction and wear behavior of 20CrMnTi needs to be further improved.

## Graphical abstract

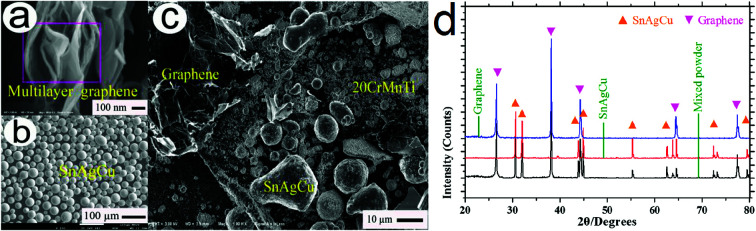

## 1. Introduction

With the heavy-duty and high-speed development of automobiles, a gear system is an indispensable element for the momentum transmission of machine components.^1–4^ 20CrMnTi steel is regarded as an excellent material for manufacturing gear systems.^5–9^ However, under some extreme conditions, such as high temperature and high vacuum, oil and grease are not sufficient to provide optimal lubrication for a 20CrMnTi-based-gear system, which is an obstacle to increasing service life and operational reliability of such a system.^10,11^ Hence, it is necessary to improve the friction and wear behavior of 20CrMnTi steel.

An effective method of improving the friction and wear behavior of 20CrMnTi is to prepare 20CrMnTi-based self-lubricating composites containing solid lubricants, such as graphene,^12–14^ carbon nanotubes (CNTs),^15,16^ and silver.^17,18^ Zhai *et al.*^12^ explored the mechanical and tribological behaviors of a Ni_3_Al-multilayer graphene composite. Their results indicated that multilayer graphene enhanced the mechanical properties of a Ni_3_Al-based composite. The observed small friction coefficient and low wear rate were mainly attributed to the intra-lamellar separation of multilayer graphene. Mallikarjuna *et al.*^15^ reported that CNTs reinforced the friction and wear behavior of the composite, and they further found that CNTs can effectively improve the anti-friction and anti-wear properties of as-prepared samples. Tronci *et al.*^17^ investigated the friction and wear behavior of silver, and found that its excellent friction and wear behavior was mainly attributed to the plastic deformation and ductility behavior of silver. However, to the best of our knowledge, few works have reported the synergetic lubrication of graphene and SnAgCu for improving the friction and wear properties of 20CrMnTi steel.

In this study, to better study the coordinating lubrication of graphene and SnAgCu, 20CrMnTi, 20CrMnTi-1.50 wt% graphene (20-Gr), 20CrMnTi-15.00 wt% SnAgCu (20-Sn), and 20CrMnTi-15.00 wt% SnAgCu-1.50 wt% graphene (20-Gr-Sn) samples were prepared by spark plasma sintering. Using a HT-1000 ball-on-disk tribometer, the tribological properties of as-prepared samples sliding against GCr15 balls were measured according to the ASTM Standard of G99-95.^19^ With the help of an electron probe microanalyzer (EPMA), the main wear mechanism was investigated by analyzing wear-scar morphology. Field-emission scanning electron microscopy (FESEM) was adopted to observe the cross-sectional morphology of wear scars. The main element contents in wear-scar cross-sections were tested using energy-dispersive spectroscopy (EDS), and X-ray photoelectron spectroscopy (XPS) was used to analyze the phase components on the wear scars.

## 2. Experimental details

### 2.1 Material preparation

The starting powders (wt%) of 0.34 Si, 1.22 Cr, 0.96 Mn, 0.13 Ti, and 97.35 Fe were mechanically mixed for 50 min by the vibration milling at a frequency of 55 Hz. Table 1 lists the main parameters of commercial powders for preparing 20CrMnTi-based material. As can be seen from the table, the powder purity used in this study exceeded 97.50%, and the powder size was mainly distributed in the region 0–25 μm. A 20CrMnTi sample (30 mm in diameter and 20 mm high) was prepared in a cylindrical graphite mold measuring 30 mm in inner diameter on the spark plasma sintering (SPS) on a D. R. Sinter® SPS3.20 system. Under the protective environment of Ar gas, the heating rate, sintering temperature, fabrication time, and holding pressure were chosen as 95–112 °C min^−1^, 950−1152 °C, 10–20 min and 30–35 MPa, respectively.

**Table 1:** Main parameters of commercial powder for preparing 20CrMnTi-based material

Starting powders	Graphene	SnAgCu	Fe	Cr	Mn	Ti	Si
Purity (%)	≥99.50	≥99.50	≥99.50	≥99.00	≥97.50	≥98.50	≥99.00
Size (nm μm^−1^)	≤20 nm	≤20 μm	≤15 μm	≤20 μm	≤15 μm	≤25 μm	≤25 μm

Table 2 shows the main components of 20CrMnTi-based composites. As can be seen from the table, approximately 15 wt% SnAgCu and 1.50 wt% graphene were chosen to prepare the 20-Gr, 20-Sn, and 20-Gr-Sn composites. The commercial multilayer graphene (5–20 nm thick and 0.5–20 μm in lateral dimension) and spherical SnAgCu powder (less than 20 μm in diameter) were purchased from Nanjing XFNANO Materials Tech Co., Ltd. Table 3 shows the main elemental content of spherical SnAgCu powder. As shown in the table, approximately 93.70 wt% Sn, 3.50 wt% Ag, and 2.80 wt% Cu were contained in spherical SnAgCu powder.

**Table 2:** Main components of 20CrMnTi-based composites

Samples	Lubricant addition contents (wt%)		20CrMnTi based material (wt%)				
	Graphene	SnAgCu	Fe	Cr	Mn	Ti	Si
20-Gr	1.50	—	97.35	1.22	0.96	0.13	0.34
20-Sn	—	15.00	97.35	1.22	0.96	0.13	0.34
20-Gr-Sn	1.50	15.00	97.35	1.22	0.96	0.13	0.34

**Table 3:** Main elemental content of spherical SnAgCu powder

Main elements	Sn	Ag	Cu
Content (wt%)	93.70 ± 0.32	3.50 ± 0.23	2.80 ± 0.14

**Fig. 1:**
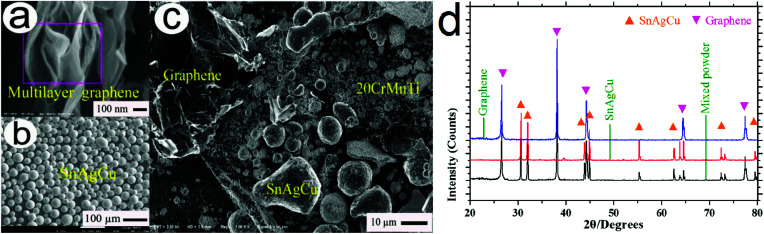
Typical FESEM morphologies (a–c) and XRD pattern (d) of multilayer graphene, SnAgCu and 20-Gr-Sn based powder.

Using a FESEM tested instrument, the typical morphologies of multilayer graphene, SnAgCu and 20-Gr-Sn based powder are shown in Fig. 1a, b, and c, respectively. The powder phase compositions are examined at the scanning speed of 0.01 ° s^−1^ using an X-ray diffractometer (XRD) with Cu Kα radiation. The results are exhibited in Fig. 1d. As shown in Fig. 1d, the main XRD peaks indicate that graphene and SnAgCu of high purity were used to obtain 20Gr-Sn based powder using a vibration milling. As shown in Fig. 1c and d, multilayer graphene and SnAgCu dispersed homogeneously in 20Gr-Sn based powder.

### 2.2 Vicker's microhardness and density

Table 4 shows the main compositions and mechanical properties of the as-prepared samples. In accordance with the ASTM standard no. E92-82,^20^ the Vickers hardness values of the as-prepared samples were measured using a HVS-1000 hardness tester, and the results are listed in Table 4. According to ASTM standard no. B962-08 and Archimedes' principle,^21^ the densities of the 20CrMnTi, 20-Gr, 20-Sn, and 20-Gr-Sn samples were measured, and the results are listed in Table 4.

**Table 4:** Main compositions and mechanical properties of as-prepared samples

Samples	Compositions (wt%)	Density (g cm^−3^)	Hardness (GPa)
20CrMnTi	0.34 Si, 1.22 Cr, 0.96 Mn, 0.13 Ti and 97.35 Fe	7.86 ± 0.11	5.22 ± 0.03
20-Gr	20CrMnTi-1.50 wt% graphene	7.21 ± 0.15	5.86 ± 0.04
20-Sn	20CrMnTi-15.00 wt% SnAgCu	7.78 ± 0.13	5.55 ± 0.05
20-Gr-Sn	20CrMnTi-1.50 wt% graphene-15.00 wt% SnAgCu	7.54 ± 0.12	5.22 ± 0.06

### 2.3 Friction and wear measurement

**Fig. 2:**
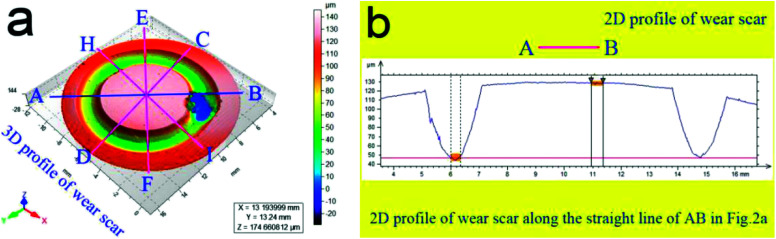
Typical wear-scar morphology of as-prepared sample: 3D (a) and 2D (b) wear-scar profiles.

According to the ASTM standard no. G99-95, at 5 N-0.2 m s^−1^, 10 N-0.4 m s^−1^, 15 N-0.6 m s^−1^, and 20 N-0.8 m s^−1^, the tribological behaviors of the as-prepared samples (30 mm in diameter and 20 mm in height) sliding against GCr15 balls (6 mm in diameter) were evaluated using the HT-1000 ball-on-disk tribometer. Before being measured, the sample surfaces were mechanically polished using emery papers of less than 1200 grits. The rotated disks of the as-prepared samples were cleaned by liquid acetone and dried using hot air. The friction radius in this study was chosen as 4.5 mm. At a relative humidity of 50–70%, the friction coefficients of the as-prepared samples were continuously recorded by the HT1000 computer-controlled system. During the friction and wear process, the wear rate *W* was defined by *W* = *U*/(*P*·*L*) = (*A*·*Q*)/(*F*·*C*).^19^ Herein, *C*, *P*, and *U* denote the sliding distance in mm, applied load in *N*, and wear volume in mm^3^, respectively. *Q* denotes the circle perimeter of wear scar in mm. *A* is the mean cross-section area of wear scar in mm^2^, which can be calculated using a surface profiler (ST400, Nanovea Corp., USA). Fig. 2 shows the typical wear-scar morphology of the as-prepared samples. As can be seen from the figure, when the test stylus of the ST400 profiler slowly moved across the wear scar along the measured line AB (see Fig. 2a), the coordinate positions of the test stylus were continuously recorded to form a two-dimensional (2D) profile of the wear scar (see Fig. 2b). Similarly, other measurements along the lines CD, EF, and HI were also carried out. After carrying out the sliding wear measurements, the mean cross-sectional area *A* of the wear scar was obtained to calculate the wear rate *W*.

## 3. Result and discussion

### 3.1 Phase research and element distribution

**Fig. 3:**
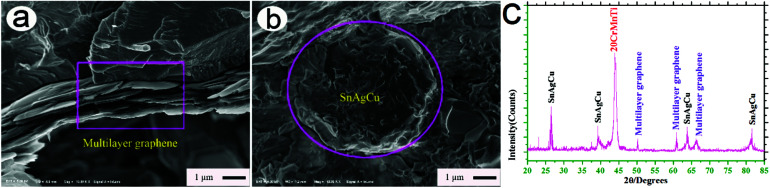
Typical FESEM cross-section morphologies of 20-Gr (a) and 20-Sn (b), as well as the XRD pattern of 20-Gr-Sn sample prepared by SPS (c).

Fig. 3a and b show typical FESEM cross-section morphologies of 20-Gr and 20-Sn. As shown in Fig. 3a, multilayer graphene was tightly embedded in 20-Gr. The multilayer morphology of graphene is well exhibited in the rectangular region of Fig. 3a. As shown in Fig. 3b, spherical SnAgCu was well combined with 20-Sn-based composite. Fig. 3c shows a typical XRD pattern of the 20-Gr-Sn sample prepared by SPS. As can be seen from Fig. 3c, the phase compositions of 20-Gr-Sn are mainly composed of 20CrMnTi, SnAgCu, and graphene according to the XRD intensities of the main diffraction peaks.

### 3.2 Analysis of friction coefficients and wear rates

**Fig. 4:**
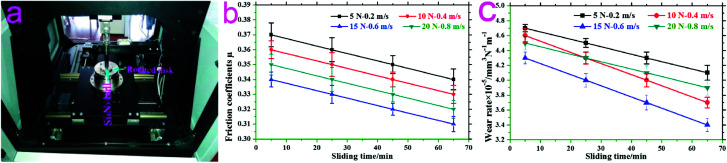
Schematic of matching samples/ball tribo-pairs (a), friction coefficients (b), and wear rates (c) of 20CrMnTi samples obtained under different test conditions.

Fig. 4a is a schematic of sample/ball tribo-pairs. As shown in Fig. 4a, the as-prepared samples were driven to slide against a fixed GCr15 ball on the high-temperature, ball-on-disk HT-1000 tribometer. The measured friction and wear behaviors of 20CrMnTi under different test conditions are shown in Fig. 4b and c. As exhibited in the figures, the tribological behavior of 20CrMnTi was significantly better at 15 N-0.6 m s^−1^ than those at 5 N-0.2 m s^−1^, 10 N-0.4 m s^−1^, and 20 N-0.8 m s^−1^.

**Fig. 5:**
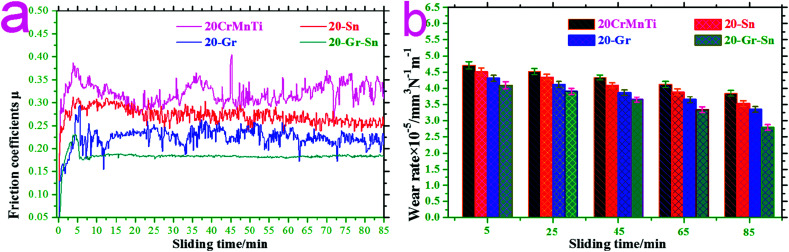
Typical friction coefficients (a) and wear rates (b) of as-prepared samples at 15 N-0.6 m s^−1^.

Fig. 5 shows the typical friction coefficients and wear rates of the as-prepared samples at 15 N-0.6 m s^−1^. As can be seen from the figure, during the sliding wear process of 0–85 min, the friction coefficients and wear rates of 20-Gr-Sn were smaller than those of 20CrMnTi, 20-Gr, and 20-Sn.

In order to further study the influence of graphene and SnAgCu on the tribological behavior of 20CrMnTi, the wear-scar morphologies of 20CrMnTi, 20-Gr, 20-Sn and 20-Gr-Sn must be investigated in detail.

### 3.3 Analysis of wear-scar morphology

**Fig. 6:**
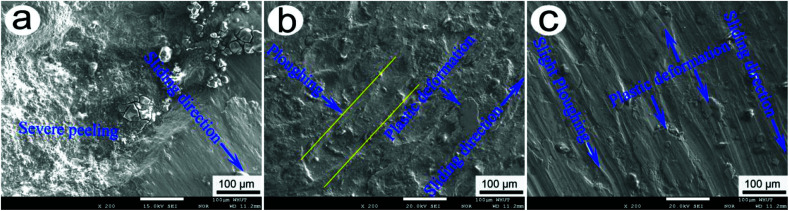
Typical EPMA morphologies of wear scars of 20CrMnTi, 20-Gr, and 20-Sn samples at 85 min.

Fig. 6 shows the typical EPMA morphologies of wear scars of 20CrMnTi, 20-Gr, and 20-Sn at 85 min. As shown in Fig. 6a, large peeling bits appeared on the wear scars, indicating that the main wear mechanism of 20CrMnTi is severe peeling. As shown in Fig. 6b, large ploughing and plastic deformation bodies formed on the wear scars, indicating that the main wear mechanisms of 20-Gr were ploughing and plastic deformation. As shown in Fig. 6c, slight ploughing and small plastic deformation bodies exist on the wear scars at 85 min. Therefore, the main wear mechanisms of 20-Sn were determined to be plastic deformation and slight ploughing. According to Yang *et al.*,^22^ if compared to those of 20CrMnTi and 20-Gr (see Fig. 6a and b), the main wear mechanisms of plastic deformation and slight ploughing (see Fig. 6c) were more helpful to realizing a low friction coefficient and small wear rate for 20-Sn.

**Fig. 7:**
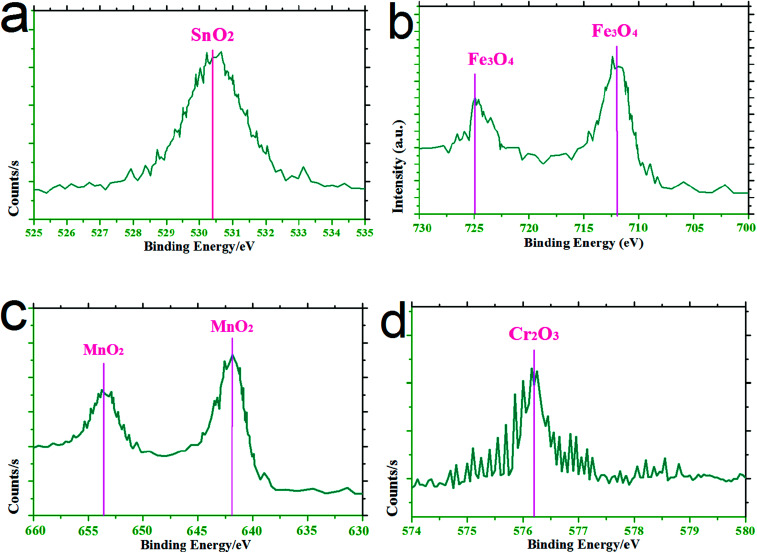
Typical XPS phase characterization of the wear scar of 20CrMnTi at 85 min.

Fig. 7 shows the typical XPS phase characterization of the wear scar of 20CrMnTi at 85 min. As shown in the figure, with reference to the results of Zhang *et al.*,^23^ Fan *et al.*,^24^ Huang *et al.*,^25^ and Yang *et al.*,^26^ the main XPS diffraction peaks were mainly attributed to the metal oxides of SnO_2_, Fe_3_O_4_, MnO_2_, and Cr_2_O_3_. When sliding wear was carried out up to 85 min, metal oxides such as SnO_2_, Fe_3_O_4_, MnO_2_, and Cr_2_O_3_ formed on wear scars, leading to a friction coefficient of approximately 0.32 and a wear rate of approximately 4.23 × 10^−5^ mm^3^ N^−1^ m^−1^ (see Fig. 5).

**Fig. 8:**
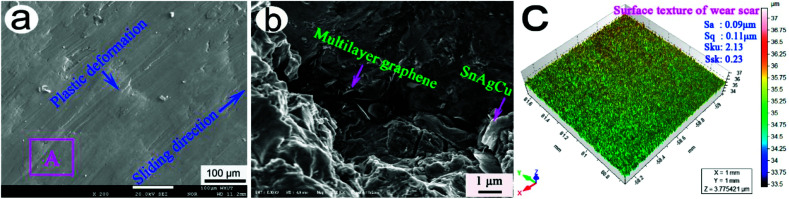
Typical EPMA morphology of wear scar of 20-Gr-Sn (a), FESEM morphology of wear scar marked by rectangle A in a (b), and texture structure of wear scar at 85 min (c).

Fig. 8a shows the typical EPMA morphology of the wear scar of 20-Gr-Sn. As shown in the figure, a plastic deformation body formed on the smooth wear scar at 85 min, indicating that the main wear mechanism of 20-Gr-Sn is plastic deformation. Fig. 8b shows the typical FESEM morphology of the wear scar marked by rectangle A in Fig. 8a. As shown in Fig. 8b, graphene and SnAgCu appear on the wear scar.

According to the discussions of Yang *et al.*,^27^ the surface texture of wear scars could be well evaluated using the height parameters of arithmetical mean height (*S*_a_), root-mean-square height (*S*_q_), height-distribution kurtosis (*S*_ku_), and height-distribution skewness (*S*_sk_). A ST400 surface profiler was used to characterize the surface texture of the wear scars. The square region (0.1 mm long) on the wear scar was measured at a scanning step length of 0.001 mm. A scanning time of approximately 20 min, the non-contact measurement scanning mode, and the scanning principle of the chromatic aberration of the white-light axis were used in this study. Fig. 8c shows the typical texture structure of a wear scar at 85 min. As can be seen from the figure, the small height parameters (*S*_a_, 0.09 μm; *S*_q_, 0.11 μm; *S*_ku_, 2.13; and *S*_sk_, 0.23) indicate that the wear-scar morphology of 20-Gr-Sn is smooth according to the definition of Yang *et al.*^27,28^

**Fig. 9:**
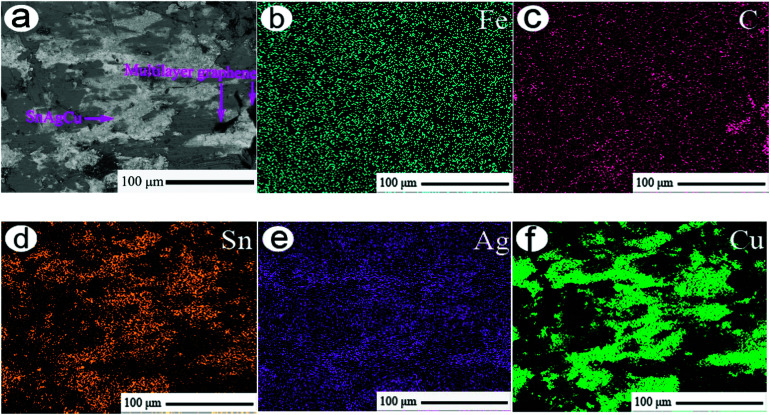
Representative EPMA back-scattering morphology (a) and main element distributions (b–f) on wear scar of 20-Gr-Sn at 85 min.

Fig. 9 shows the representative EPMA back-scattering morphology and main element distributions on the wear scar of 20-Gr-Sn at 85 min. As shown in the figure, at 85 min multilayer graphene and SnAgCu are uniformly distributed on the wear scar.

Hence, massive SnAgCu appeared on the wear scar to undergo the significant plastic deformation at the repeating effect of friction force in Fig. 8a and b. The plastic deformation effectively repaired the wear scar to form a well-textured structure (see Fig. 8c). In addition, multilayer graphene possessed small intra-la adherence. During the process of sliding wear, graphene interlamination separation effectively prevented the surface material from being destroyed, resulting in low wear rate. The small shearing strength of SnAgCu is helpful in lowering the friction force, which is beneficial to the realizing a small friction coefficient. Hence, the excellent synergistic lubrication of graphene and SnAgCu caused 20-Gr-Sn to exhibit a small friction coefficient and a lower wear rate.

### 3.4 Analysis of wear-scar cross-sections

**Fig. 10:**
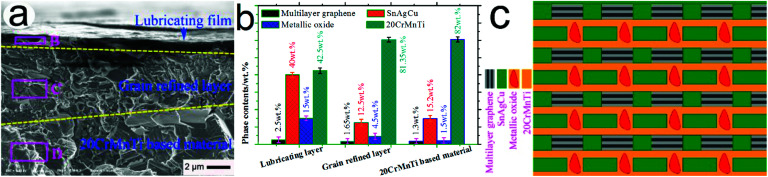
Typical FESEM cross-sectional morphology of wear scar of 20-Gr-Sn (a); main element content in the rectangular regions B, C, and D in a (b); and schematic of lubrication-film structure (c) at 85 min.

Fig. 10a shows the typical FESEM cross-sectional morphology of the wear scar of 20-Gr-Sn at 85 min. As can be seen from the figure, the typical stratification structures existed under the wear-scar subsurface, and were mainly composed of lubrication film, a grain-refined layer, and 20CrMnTi-based material. Fig. 10b shows the main elemental content in the rectangular regions of B, C and D (see Fig. 10a). As shown in Fig. 10a and b, approximately 2.5 wt% graphene, 40 wt% SnAgCu, and 15 wt% metal oxides exist in the lubrication film. Graphene, SnAgCu, and O contents in the grain-refined layer were approximately 1.65 wt%, 12.5 wt%, and 4.5 wt%, respectively. Fig. 10c is a schematic of the lubrication film structure. As can be seen in the figure, graphene, SnAgCu, and metal oxides appear in the lubrication film, and were tightly combined with the 20CrMnTi-based material, leading to the formation of the lubrication film. In accordance with Zhai *et al.*,^29^ a lubrication film with graphene, SnAgCu, and metal oxides resulted in a small friction coefficient and low wear rate.

**Fig. 11:**
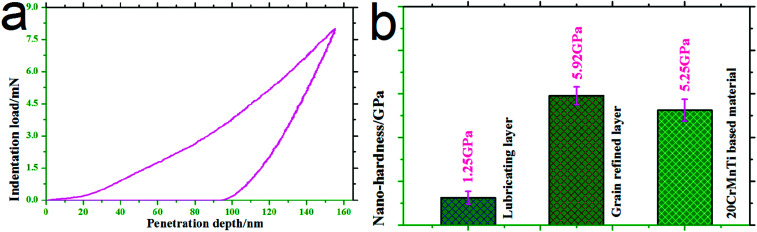
Typical nano-indentation curve (a) and nano-indentation hardness (b) in wear scar cross-sections at 85 min.

Fig. 11 shows the typical nano-indentation curve and nano-indentation hardness in wear-scar cross-sections at 85 min. As shown in the figure, after being tested, a massive amount of material, 40 wt% SnAgCu, existed in the lubrication film (see Fig. 10b), and the indentation hardness of the lubrication film was approximately 1.25 GPa. Indentation hardness values of approximately 5.92 GPa for the grain-refined layer and approximately 5.25 GPa for 20CrMnTi-based material were obtained. During the friction and wear process of 0–85 min, a slight amount of graphene (2.5 wt%) was gradually exposed to the wear scar, which was tightly combined with SnAgCu, leading to the formation of a lubrication film. The lubrication film possessed a low hardness of 1.25 GPa, and existed on the grain-refined layer with a high hardness of 5.92 GPa. According to the ones proposed by Zhai *et al.*,^29^ these values facilitated a low friction coefficient and low wear rate.

### 3.5 Analysis of GCr15 ball

**Fig. 12:**
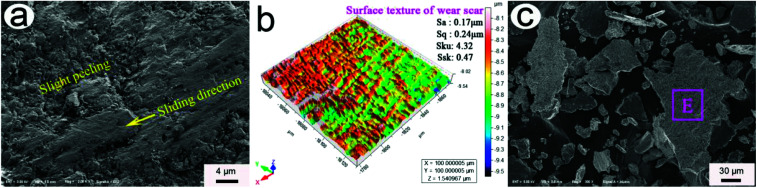
Typical FESEM morphology of GCr15 ball (a), texture structure of wear scar (b), and FESEM morphology of wear debris (c) at 85 min.

Fig. 12a and b show the typical FESEM morphology of a GCr15 ball and the texture structure of its wear scar at 85 min. As can be seen from the figure, slight peeling bits formed on the wear scar of GCr15 ball, indicating that the main wear mechanism was slight peeling. The low height parameters (*S*_a_, 0.17 μm; *S*_q_, 0.24 μm; *S*_ku_, 4.32; and *S*_sk_, 0.47) were obtained, indicating that the wear scar of the GCr15 ball was smooth. Fig. 12c shows the typical FESEM morphology of the wear debris at 85 min. As shown in the figure, the main structure of the wear debris was in the form of a large sheet. The main element contents (wt%) in the rectangle E of the wear debris in the figure were about 1.86 graphene–24.72 Sn–0.82 Ag–0.67 Cu–62.32 Fe–1.18 Cr–1.02 Mn–0.17 Ti–7.24 O. Graphene and SnAgCu appeared on the wear scar of the GCr15 ball, indicating that graphene and SnAgCu were transferred to the matching-pair friction interface. This effectively lowered the friction resistance and material loss of 20-Gr-Sn, which greatly facilitated to realizing a small friction coefficient and low wear rate.

## 4. Conclusions

In this paper, we mainly explored the coordinating lubrication of graphene and SnAgCu, reaching the following conclusions.

(1) At 0–85 min, a smaller friction coefficient and lower wear rate were obtained for 20-Gr-Sn compared to those of 20CrMnTi, 20-Gr, and 20-Sn samples.

(2) A massive amount of both graphene and SnAgCu existed on the smooth wear scar of 20-Gr-Sn, exhibiting excellent friction and wear properties, leading to a low friction coefficient and low wear rate.

(3) Graphene, SnAgCu, and metal oxides appeared on the wear scars of as-prepared samples, and combined with 20CrMnTi-based material, resulted in the formation of a lubrication film.

(4) A lubrication film (1.25 GPa in hardness) existed on the high-hardness grain-refined layer (5.92 GPa), which facilitated a low friction coefficient and low wear rate.

## Conflicts of interest

There are no conflicts to declare.

## Supplementary Material
